# Co-Encapsulation of Tannic Acid and Resveratrol in Zein/Pectin Nanoparticles: Stability, Antioxidant Activity, and Bioaccessibility

**DOI:** 10.3390/foods11213478

**Published:** 2022-11-02

**Authors:** Xiao Liang, Wanting Cheng, Zhanhong Liang, Yiling Zhan, David Julian McClements, Kun Hu

**Affiliations:** 1 Food Science School, Guangdong Pharmaceutical University, Zhongshan 528458, China; 2 Clinical Medicine Department, Guangdong Maoming Health Vocational College, Maoming 525400, China; 3 Department of Food Science, University of Massachusetts, Amherst, MA 01003, USA

**Keywords:** zein nanoparticles, tannic acid, resveratrol, co-encapsulation, bioaccessibility, antioxidant activity

## Abstract

Hydrophilic tannic acid and hydrophobic resveratrol were successfully co-encapsulated in zein nanoparticles prepared using antisolvent precipitation and then coated with pectin by electrostatic deposition. The encapsulation efficiencies of the tannic acid and resveratrol were 51.5 ± 1.9% and 77.2 ± 3.2%, respectively. The co-encapsulated nanoparticles were stable against aggregation at the investigated pH range of 2.0 to 8.0 when heated at 80 °C for 2 h and when the NaCl concentration was below 50 mM. The co-encapsulated tannic acid and resveratrol exhibited stronger in vitro antioxidant activity than ascorbic acid, as determined by 1,1-diphenyl-2-picrylhydrazyl free radical (DPPH·) and 2,2′-azinobis (3-ethylberizothiazoline-6-sulfonic acid) radical cation (ABTS^+^·) scavenging assays. The polyphenols-loaded nanoparticles significantly decreased the malondialdehyde (MDA) concentration and increased the superoxide dismutase (SOD) and catalase (CAT) activities in peroxide-treated human hepatoma cells (HepG2). An in vitro digestion model was used to study the gastrointestinal fate of the nanoparticles. In the stomach, encapsulation inhibited tannic acid release, but promoted resveratrol release. However, in the small intestine, it led to a relatively high bioaccessibility of 76% and 100% for resveratrol and tannic acid, respectively. These results suggest that pectin-coated zein nanoparticles have the potential for the co-encapsulation of both polar and nonpolar nutraceuticals or drugs.

## 1. Introduction

Over the past decade or so, there has been a great deal of interest in developing colloidal delivery systems that can encapsulate, protect, and release bioactive substances, such as drugs, nutrients, and nutraceuticals [1,2,3]. To be suitable for commercial applications, these delivery systems should be fabricated from affordable and label-friendly food ingredients using cost-effective and sustainable manufacturing operations that are capable of being scaled up [4]. For these reasons, hydrophobic proteins have attracted considerable attention as potential building blocks for constructing edible colloidal delivery systems. Zein is the major storage protein of corn [4], which has been classified as a generally recognized as safe ingredient by the United States Food and Drug Administration [5]. Over half of the amino acids in zein are hydrophobic, which make it dissolvable in aqueous ethanol solutions (60–90%, *v*/*v*), but not in water [6]. This differential solubility characteristic can be utilized to fabricate nutraceutical-loaded nanoparticles from zein using an antisolvent precipitation approach. In this approach, the zein and nutraceuticals are first dissolved in a concentrated ethanol solution. This solution is then injected into water, which serves as an antisolvent, promoting the self-assembly of the protein and nutraceutical molecules into nanoparticles. Many kinds of nutraceuticals and other bioactive agents can be dissolved in ethanol solutions and therefore, encapsulated into zein nanoparticles, including curcumin, resveratrol, quercetin, and thymol [7,8,9,10]. Encapsulation of these substances often improves their water dispersibility, chemical stability, bioaccessibility, and bioactivity.

One of the main challenges with using zein nanoparticles is that they have a relatively high surface hydrophobicity, which increases their tendency to aggregate with each other in aqueous solutions. This problem can be overcome by adding emulsifiers, such as Tween 80 [11], sodium caseinate [12], or *β*-lactoglobulin [13], that adsorb to the zein nanoparticle surfaces and reduce the surface hydrophobicity. Charged polysaccharides that attach to the surfaces of the zein nanoparticles and increase the electrostatic and steric repulsion between them, such as pectin [14], alginate [4], gum arabic [15], and chitosan [16], can also be added. Consequently, the stability and functional performance of zein nanoparticles can be improved by using appropriate emulsifiers or polysaccharides.

A potential advantage of zein nanoparticles as delivery systems for some applications is that they can co-encapsulate two or more bioactive agents, which can lead to enhanced activity [17]. Co-encapsulation is achieved by including several bioactive agents in the ethanol solution used to prepare the nanoparticles via antisolvent precipitation [9]. For instance, curcumin and piperine have been co-encapsulated in carrageenan-coated zein nanoparticles, which improved their resistance to degradation by heat and light, as well as controlled their release under simulated gastrointestinal conditions [18]. The introduction of nutraceuticals can affect the size and charge of protein nanoparticles. For example, the introduction of *α*-tocopherol and resveratrol was shown to increase the mean particle diameter and ζ-potential of zein nanoparticles [9]. Consequently, it is important to consider the potential impact of nutraceutical incorporation on nanoparticle formation. 

The polarity of nutraceuticals also affects their encapsulation and performance in nanoparticle-based delivery systems [19]. Previously, researchers have mainly focused on the co-encapsulation of hydrophobic compounds, such as curcumin, resveratrol, rutin, piperine, coenzyme-Q10, and vitamin D [20,21,22,23]. There have been few previous reports on the co-encapsulation of polar and nonpolar compounds. 

**Figure 1:**
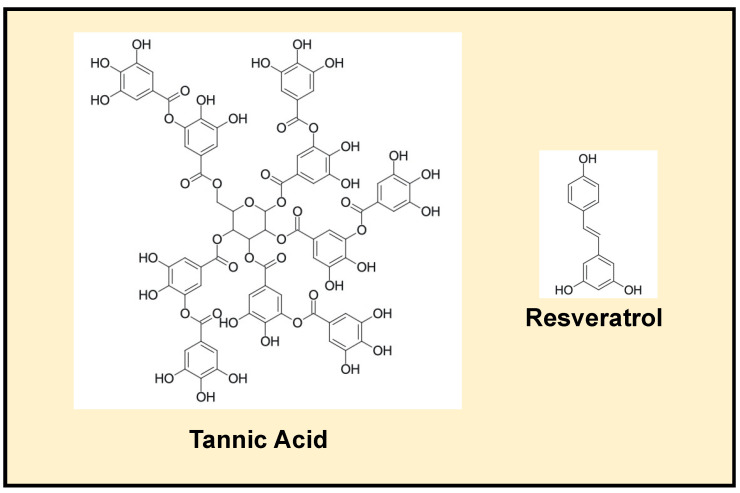
Structures of tannic acid and resveratrol.

Tannic acid (TA) is a relatively polar polyphenol with a high water solubility because it contains numerous hydroxyl groups (Figure 1). Commercially, TA is not molecularly homogenous. Typically, it consists of a mixture of substances that exhibits a central glucose molecule with a number (2–12) of branched galloyl moieties attached [24]. This structure of TA means that many of its phenolic groups are available to interact with other molecules, such as proteins, polysaccharides, and alkaloids [25,26]. TA has been reported to exhibit multiple biological behaviors, including antitumor, antimicrobial, antimutagen, and antioxidant activities [24,27,28]. Moreover, it is also generally recognized as safe by the Food and Drug Administration. Much concern has been focused on the biopolymer crosslinking effect of TA, such as polysaccharide TA-based or peptide TA-based hydrogels [29]. TA-chitosan conjugates also exhibited superior antioxidant activity [30]. There were very few reports regarding the use of a delivery system to control TA release during digestion [28]. Resveratrol is a relatively non-polar polyphenol that has a lower water-solubility than tannic acid because it has fewer hydroxyl groups (Figure 1). Resveratrol is found in appreciable levels in some edible fruits and nuts, including blueberries, grapes, and peanuts. It has been reported to possess anti-inflammation, antioxidant, anti-aging, hepato-protective, neuro-protective, and cardio-protective activities [31]. Consequently, it is being explored for its potential application as a nutraceutical ingredient in functional foods and drugs. However, its poor solubility in water has limited the commercial application of resveratrol. Nano-delivery systems, such as liposomes [32,33], protein nanoparticles [34,35], nanoemulsions [36], cyclodextrin [37], etc., were developed to prevent instability due to environmental stress (light, heat, oxygen, etc.) and increase water solubility, bioaccessibility, and bioactivity.

In previous studies, we developed pectin-coated zein nanoparticles that could be used as delivery system for bioactive agents [31,38,39]. We showed that the encapsulation of hydrophobic nutraceuticals (curcumin or resveratrol) in these delivery systems could increase their antioxidant activity, anti-inflammatory activity, bioaccessibility, and cellular uptake. We also used this delivery system to encapsulate tannic acid to control TA releasing during gastrointestinal digestion [28], without affecting its antioxidant capacity. In the current study, we investigated the ability of these core-shell nanoparticles to co-encapsulate a hydrophilic (tannic acid) and a hydrophobic (resveratrol) nutraceutical. Moreover, we evaluated the impact of encapsulation on the in vitro antioxidant activity and bioaccessibility of the co-encapsulated polyphenols using a simulated gastrointestinal model. The results of this study may facilitate the design of colloidal delivery systems for the co-encapsulation of polar and nonpolar bioactive ingredients.

## 2. Materials and Methods 

### 2.1. Materials

Zein (>98%), tannic acid (>99.9%), pectin extracted from citrus peel (galacturonic acid ≥74.0% on a dried basis; moisture ≤ 10%), bile salt (>95%), pancreatin (activity, 8×USP), and pepsin from porcine gastric mucosa (activity, 780 U/mg) were purchased from Sigma-Aldrich (St Louis, MO, USA). Resveratrol (>98%) was purchased from Adamas-Beta (Shanghai, China). Fetal bovine serum (FBS), penicillin streptomycin double antibody solution, and trypsin were obtained from Hyclone (Logan, UT, USA). Dulbecco’s modified eagle medium (DMEM) and sterile dimethyl sulfoxide (DMSO) were purchased from TheromFisher. Malondialdehyde (MDA), superoxide dismutase (SOD), and catalase (CAT) test kits were purchased from Nanjing Built Bioengineering (China). Other chemicals were of analytical grade.

### 2.2. Preparation of Nutraceutical-Loaded Nanoparticles 

The co-encapsulated pectin-coated nanoparticles were prepared according to our previous method [38,39], with some slight modifications: Briefly, 1.0 g of zein was solubilized in 50.0 mL of aqueous ethanol solution (85%, *v*/*v*) with magnetic stirring at 500 rpm for 30 min (R05, IKA Inc., Staufen, Germany). Then weighed amounts of tannic acid (0.1 g) and resveratrol (0.12 g) were added into the zein solution, and it was continuously stirred in the dark for another 60 min. A zein: tannic acid: resveratrol weight ratio of 10:1:1.2 was selected for our studies, which was based on preliminary experiments that showed this composition could produce nanoparticles with a high loading efficiency and small size. Then, 4.0 mL of the tannic acid–resveratrol–zein solution was injected into 16 mL of double distilled water (adjusted to pH 4.0 with 0.1 M HCl) through a syringe and continually stirred at 900 rpm for 5 min. The ethanol in the prepared nanoparticle dispersion was evaporated with a rotary evaporator (RE-2000, Yarong Biochemical Instrument, Shanghai, China) at 40 °C, and an appropriate volume of pH 4.0 water was added until the final volume of the dispersion was 20 mL [38]. The resulting nanoparticle dispersion was then mixed with 20.0 mL of pectin solution (0.11% *w*/*v*, pH 4.0), with continuous stirring at 900 rpm for 10 min. The sample was then centrifuged at 3000 rpm for 10 min to remove any large particles. 

### 2.3. Particle Size and Zeta Potential Measurements

The mean particle diameter, polydispersity index (PDI), and surface potential (ζ-potential) of the nanoparticle dispersions were measured at 25 °C using a dynamic light scattering and electrophoresis instrument (Nano-ZS 90, Malvern Instruments, Malven, UK), as reported previously [39]. The samples were diluted with acidified water (pH 4.0) before analysis to avoid multiple scattering effects [34].

### 2.4. Field Emission Scanning Electron Microscopy (SEM)

The nanoparticle dispersions were freeze-dried, and the resulting powders were fixed to a sample holder and then sputter-coated with gold. The microstructures of the samples were then characterized using a field emission scanning electron microscope operating at an accelerating voltage of 10.0 kV (NovaNanoSEM 430, FEI, Eindhoven, The Netherlands). 

### 2.5. Nutraceutical Encapsulation

Freshly prepared nanoparticle dispersions were centrifuge-filtered with a 10 kD membrane filter (4000 rpm, 20 min) to separate non-encapsulated nutraceuticals (in the filtrate).

For tannic acid analysis, the filtrate was diluted with DMSO, and then the absorbance of the resulting solution was determined using an ultraviolet (UV)-visible spectrometer (UV-1800, SHIMADZU, Japan) at 278 nm. The mass of tannic acid in the filtrate was calculated based on a calibration curve prepared using tannic acid solutions with concentrations of 0 to 20 µg/mL (R^2^ = 0.999). The tannic acid loading efficiency was then calculated:

(1) $$\mathrm{Tannic}\;\mathrm{acid}\;\mathrm{loading}\;\mathrm{efficiency}\left(\%\right)=\frac{M_{t}-M_{f}}{M_{t}}\times 100\%$$

Here, $M_{f}$ and $M_{t}$ are the masses of tannic acid in the filtrate and in the total system, respectively.

The resveratrol loading efficiency was determined using a high performance liquid chromatography (HPLC) method previously described, with some modifications [39]: Briefly, freeze-dried nanoparticles (solubilized in DMSO) were diluted with a mobile phase (methanol:ultrapure water = 50:50, *v*/*v*) and then filtered through a 0.2 μm membrane. Then, 10 µL of the filtered solution was injected into an ultrahigh performance liquid chromatography (UHPLC) system with a C18 reverse-phase column (250 mm × 4.6 mm, 5 µm, Hibar, Germany) maintained at 35 °C. The mobile phase flow rate was 1 mL/min, and the absorbance wavelength was 306 nm [39]. A calibration curve was established based on peak areas corresponding to resveratrol concentrations of 0–250 µg/mL (R^2^ = 1), and the resveratrol content was calculated. The resveratrol loading efficiency was calculated using the following equation:

(2) $$\mathrm{Resveratrol}\;\mathrm{loading}\;\mathrm{efficiency}\%=\frac{C}{C_{t}}\times 100\%$$

Here, C and $C_{t}$ are the concentrations of resveratrol remaining in the delivery system and originally added to the delivery system, respectively.

### 2.6. Nanoparticle Dispersion Stability

The stability of freshly prepared nanoparticle dispersions was determined in relation to changes that may be precipitated by environmental stresses that they may experience in foods.

#### 2.6.1. pH Stability

Freshly prepared nanoparticle dispersions were adjusted to pH 2.0 to 8.0 using HCl or NaOH solutions.

#### 2.6.2. Salt Stability

Sodium chloride was added to nanoparticle dispersions to reach final salt concentrations ranging from 0 to 100 mM NaCl.

#### 2.6.3. Heat Stability

The nanoparticle dispersions were heated in a water bath at 80 °C for periods ranging from 0 to 120 min. After heating, the samples were cooled to room temperature with tap water.

#### 2.6.4. Storage Stability

The nanoparticle dispersions were stored in a refrigerator at 4 °C for periods ranging from 0 to 28 days.

After each of these treatments, the overall appearances of the nanoparticle dispersions were recorded by a digital camera. In addition, the mean diameter, PDI, and ζ-potentials of the particles were determined using the method presented in Section 2.3.

### 2.7. In Vitro Antioxidant Activity Determination of Co-Encapsulated Nanoparticles

#### 2.7.1. DPPH·Scavenging Activity

The DPPH·scavenging capacity of the samples was measured using a previously described method [40], with some modifications. Briefly, co-encapsulated nanoparticles and other test samples were prepared at multiple concentrations. Then, 2 mL of a 100 μM DPPH·ethanol solution was mixed with an equal amount of a test sample and incubated in the dark at ambient temperature. After 30 min, the absorbance of the reaction solution was measured at 517 nm. The DPPH· scavenging percentage of the samples was calculated using the following equation:

(3) $$\mathrm{DPPH}\cdot \mathrm{Scavenging}\left(\%\right)=\frac{\mathrm{Ac}-\mathrm{As}}{\mathrm{Ac}}\times 100\%$$

Here, the Ac and As are the absorbance of the control and sample solutions, respectively. The concentration of an antioxidant (test sample) required to achieve a 50% reduction in DPPH· radical scavenging rate (SC_50_) was calculated from a standard curve established by plotting the antioxidant concentration against DPPH· scavenging percentage for each test sample [34]. The DPPH· scavenging capacity of plain nanoparticles (no encapsulation) was also determined.

#### 2.7.2. ABTS^+^· Scavenging Capacity 

The ABTS^+^· scavenging capacity of co-encapsulated polyphenols was determined using a method described previously [41,42], with some modifications. An ABTS stock solution was prepared by dissolving 7 mM ABTS in water. An ABTS radical cation solution was then prepared by mixing the ABTS stock solution and a 4.9 mM potassium persulfate solution in equal quantities and allowing them to stand at ambient temperature in the dark for16 h. The resulting ABTS^+^· solution was then diluted with PBS (10 mM, pH 7.4) to an absorbance of 0.70 (±0.02) cm^−1^ at 734 nm at 30 °C [34]. The test samples were prepared with multiple concentrations, and 40 microliter samples were dissolved in 4 mL of ABTS^+^· solutions at 30 °C in the dark. After 6 min reaction, the absorbance was measured at 734 nm. Phosphate buffer solution and absolute ethanol were used as blanks [28]. The scavenging capability of the samples was determined using the following equation:

(4) $${\;\mathrm{ABTS}}^{+}\cdot \mathrm{scavenging}\left(\%\right)=\frac{\mathrm{Ac}-\mathrm{As}}{\mathrm{Ac}}\times 100\%$$

Here, Ac and As are the absorbance of the control solution and sample, respectively. SC_50_ values were calculated from standard curves established by plotting antioxidant concentrations against ABTS^+^· scavenging percentage. The scavenging capacity of plain nanoparticles (no encapsulation) was also determined.

### 2.8. HepG2 Cell Culture and Cytotoxicity Assay

HepG2 cells were cultured in DMEM solution supplemented with 10% FBS and 1% penicillin-streptomycin solution. Cells were maintained at 37 °C under a humidified atmosphere with 5% CO_2_ in an incubator [38]. Cells at 80–90% confluence were used for the treatments.

The cell counting kit 8 (CCK8) assay method was used to evaluate cell viability, which has been previously described in detail [38]. Briefly, cells (1 × 10^4^) were seeded in 96-well microtiter plates. After 24 h, the cell culture media were aspirated, and the cells were treated with 100 μL culture medium containing various concentrations of co-encapsulation nanoparticles, tannic acid-loaded nanoparticles, resveratrol-loaded nanoparticles, or physical mixtures that contained free tannic acid and resveratrol (at the same concentrations as in the co-encapsulated nanoparticles) for another 24 h. The samples were dissolved in DMSO, while ensuring that the final concentration of DMSO in the culture solutions was below 0.1% to avoid poisoning of the HepG2 cells. Then, the cells were washed with PBS twice and incubated with 100 μL DMEM medium and 10 μL CCK8 solution for 3 h. The optical density (OD) values were determined with a microplate reader (BioTek Instruments, Winooski, VT, USA) at 450 nm [38]. The impact of plain nanoparticles on cell viability was also determined. 

### 2.9. Determination of Oxidation Markers in Peroxide-Treated HepG2 Cells

HepG2 cells were seeded in six-well plates (6 × 10^5^ cells/well) in complete medium and cultured until a uniform monolayer formed. They were then divided into three groups: a co-encapsulated nanoparticles treated group; an H_2_O_2_ treated group; and a blank control group. After being incubated for 24 h, the experimental group was treated with fresh medium containing co-encapsulated nanoparticles, while the model and blank control groups were treated with fresh medium (without samples). Then, the cells were treated with 950 μmol/L H_2_O_2_ for 4 h (except the blank control group). Finally, the cells were lysed on ice with cell lysate for 30 min, and then centrifuged at 12,000 r/min for 10 min at 4 °C to obtain the supernatant. The levels of MDA, SOD, and CAT in the cells were determined according to the instructions in the commercial test kits.

### 2.10. Simulated Gastrointestinal Digestion

An in vitro gastrointestinal tract (GIT) model was used to study the potential gastrointestinal fate and bioaccessibility of tannic acid and resveratrol in different formulations, including free resveratrol, free tannic acid, and co-encapsulated tannic acid and resveratrol. The simulated gastrointestinal digestion model used in this study was based on one described previously [39], with some modifications.

Gastric stage [43]: Test samples were dissolved in 20 mL of ultrapure water to obtain a polyphenol concentration of 0.5 mg/mL. The dispersions were then mixed with 20 mL of simulated gastric fluid (double distilled water adjusted to pH 1.2 with HCl, containing 50 mM NaCl, and 3.2 mg/mL pepsin). The resulting mixtures were then adjusted to pH 2.5 using NaOH solution and shaken continuously at 80 rpm and 37 °C in an incubator (Constant Temperature Shaker, THZ-82, Shanghai, China) for different amounts of time. After completion of the gastric digestion simulation, the samples were heated at 80 °C for 5 min in a water bath to inactive the pepsin, and double distilled water was added to compensate for any loss of water due to evaporation.

Small intestine stage: Each sample (40 mL) resulting from the gastric phase was adjusted to pH 7.0 with NaOH solution, and 0.1875 g of bile salt was added. The sample was maintained in a 37 °C water bath with continuous stirring at 100 rpm [39]. Then 0.144 g pancreatin was added to initiate digestion. An automatic titration system (902 Titrando, Metrohm, Herisau, Switzerland) was used to maintain the samples at a constant pH (pH 7.0) by the automatic addition of NaOH solution [28]. After completion of the small intestine phase, samples were heated at 90 °C for 5 min in a water bath to inactive the pancreatin, and double distilled water was added to compensate for any loss of water due to evaporation [43].

Bioaccessibility: The amount of tannic acid and resveratrol released from the various formulations was measured during the gastric and small intestine stages. For the gastric stage, tannic acid and resveratrol release were measured after the samples were incubated for 30, 60, 90, and 120 min in simulated gastric fluids, while for the small intestine phase, it was measured after the samples were incubated for 30, 60, 120, 180, and 240 min in simulated small intestinal fluids [39]. After collection, the samples were centrifuged at 5000× *g* for 30 min to collect supernatants and then diluted in DMSO to determine the amount of tannic acid or resveratrol released, according to the methods described in Section 2.5. The percentages of tannic acid and resveratrol released from the delivery systems into the digestion fluids were then calculated using the following equation:

(5) $$\mathrm{Releasing}\;\mathrm{percentage}\%=\frac{\mathrm{Mass}\;\mathrm{of}\;\mathrm{released}\;\mathrm{in}\;\mathrm{supernatant}}{\mathrm{Mass}\;\mathrm{of}\;\mathrm{tannic}\;\mathrm{acid}\;\mathrm{or}\;\mathrm{resceratrol}\;\mathrm{before}\;\mathrm{digestion}}\times 100\%$$

### 2.11. Statistical Analysis

Each experiment was repeated three times, and the results are expressed as the mean and standard deviation. Data were analyzed using SAS 9.40 statistical software. A probability of *p* < 0.05 was taken to mean that the differences between data points were statistically significant.

## 3. Results and Discussion

### 3.1. Morphology and Loading Efficiency of Co-Encapsulated Nanoparticles

**Figure 2:**
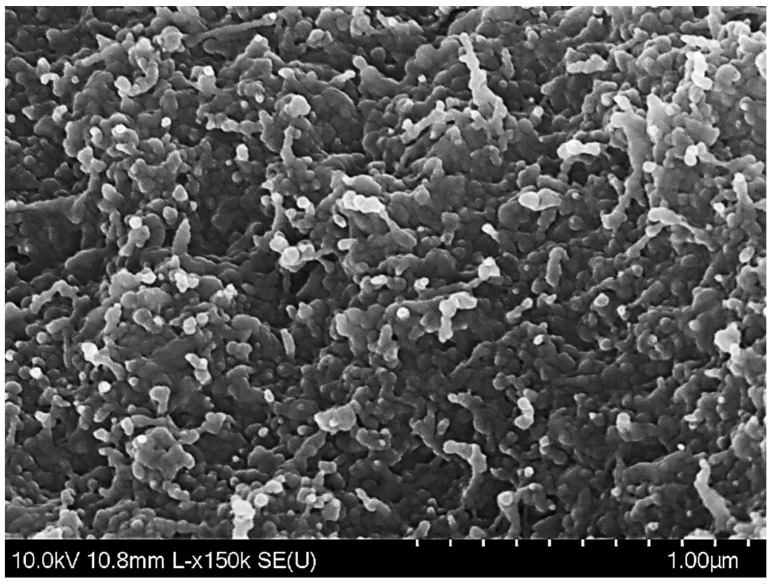
SEM image of freeze-dried nanoparticle co-loaded with tannic acid and resveratrol.

The morphology of freeze-dried co-encapsulated nanoparticles is shown in Figure 2. After lyophilization, most of the particles were clustered together and exhibited approximately elliptic or spherical shapes, with rough surfaces and diameters below 200 nm. This microstructure was different from that reported for resveratrol-loaded nanoparticles, which were predominantly spherical and had smooth surfaces [34], or tannic acid-loaded nanoparticles, which were mostly spherical, but had rough surfaces [28]. Therefore, the results of our SEM analysis suggest that the morphology and aggregation state of the zein nanoparticles was affected by the combined presence of tannic acid and resveratrol during antisolvent precipitation.

Dynamic light scattering analysis indicated that the freshly prepared nanoparticles had relatively small average particle diameters (166.8 ± 6.8 nm) and low polydispersity indices (PDI = 0.25 ± 0.01). This result suggests that fairly uniform nanoparticles were present when they were dispersed in aqueous solutions. The encapsulation efficiency of tannic acid and resveratrol in the nanoparticles were 51.5 ± 1.9% and 77.2 ± 3.2%, respectively. Our value for resveratrol when it was co-encapsulated within the zein nanoparticles was fairly similar to that reported when resveratrol was encapsulated alone within similar nanoparticles [34]. This result suggests that the presence of the tannic acid did not have a major effect on the encapsulation of the resveratrol. In contrast, resveratrol seemed to affect the encapsulation of tannic acid. In our study, the percentage of tannic acid co-encapsulated in the zein nanoparticles was relatively low compared to the value obtained when tannic acid alone was encapsulated in similar nanoparticles [28]. A possible reason for this effect was that tannic acid has a relatively high water solubility and thus may migrate into the aqueous phase during the co-precipitation process [44]. Co-encapsulated hydrophobic compounds have also been reported to have lower encapsulation efficiencies than single compounds encapsulated in similar systems. For instance, the loading efficiency of curcumin and resveratrol was only 54% and 71% when they were co-encapsulated into zein nanoparticles, respectively [45]. 

### 3.2. Particle Dispersion Stability under Environmental Stress

**Figure 3:**
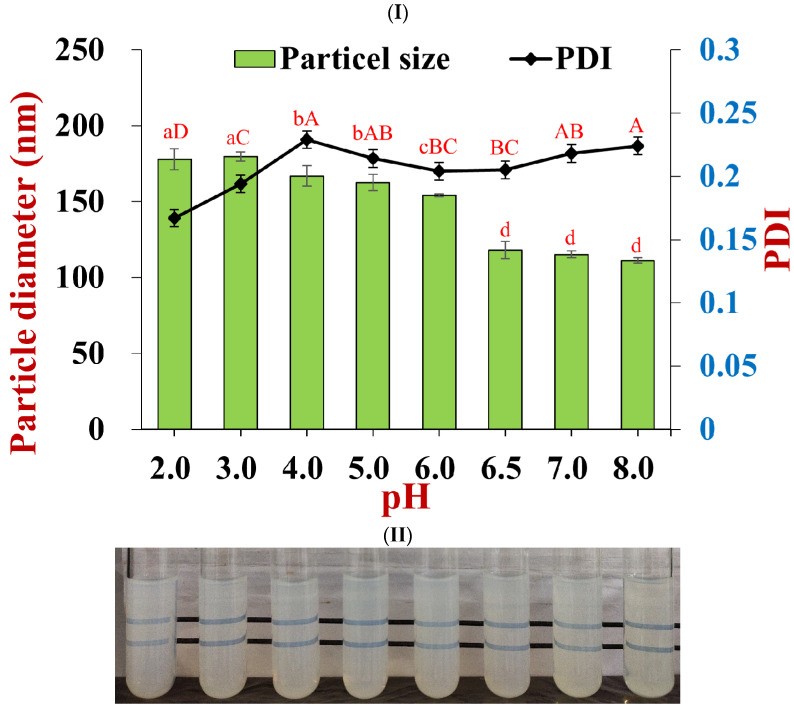
Effect of pH on the stabilities of nanoparticles co-loaded with tannic acid and resveratrol. (**I**) Particle diameter and PDI at different pH (lowercase statistic notation—particle size; capital statistic notation—PDI, *p* < 0.05); (**II**) the appearance of particles at pH values ranging from 2.0 to 8.0.

The average diameter of the nanoparticles containing the co-encapsulated nutraceuticals gradually changed from 177.9 ± 7.0 nm to 111.2 ± 1.8 nm when the pH was increased from 2.0 to 8.0 (Figure 3II). Moreover, there was a slight increase in the polydispersity of the nanoparticles from 0.17 to 0.23 across the same pH range. These results suggest that the colloidal dispersions were resistant to particle aggregation over this pH range. This finding was confirmed by visual observations of the samples, which showed that they remained transparent across this pH range (Figure 3II). It should be noted that the nutraceutical-loaded nanoparticles had a small diameter (118.1 ± 5.6 nm) at pH 6.5, which is close to the isoelectric point (pH 6.2) of pure zein [46]. This effect can partly be attributed to the electrostatic and steric repulsion generated by the pectin coating around the zein nanoparticles. However, the size of nanoparticles loaded with either resveratrol [39] or curcumin [42] has been reported to increase significantly when they were adjusted to pH 6.5. Under these pH conditions, resveratrol and curcumin are uncharged hydrophobic compounds, whereas tannic acid is an anionic hydrophilic compound. The presence of tannic acid at the surfaces of the zein nanoparticles might therefore have changed the hydrophobic and electrostatic interactions between them, thereby improving their stability around the isoelectric point of zein.

**Figure 4:**
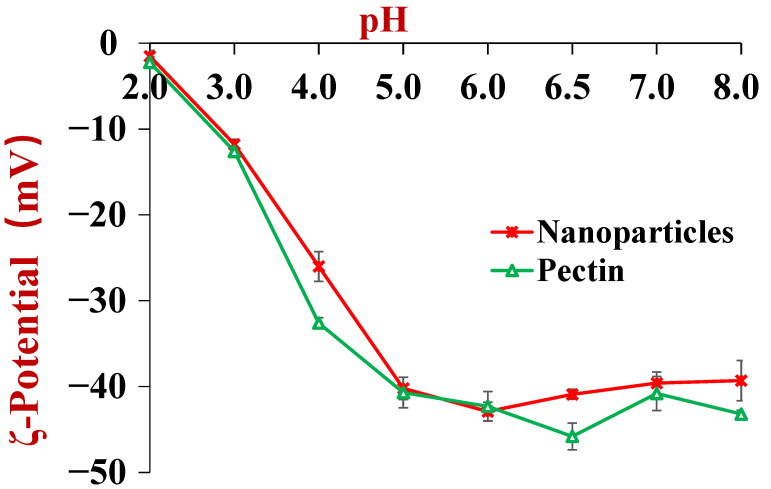
The ζ-potential of tannic acid and resveratrol co-encapsulated zein/pectin nanoparticles at pH values ranging from 2.0 to 8.0.

**Figure 5:**
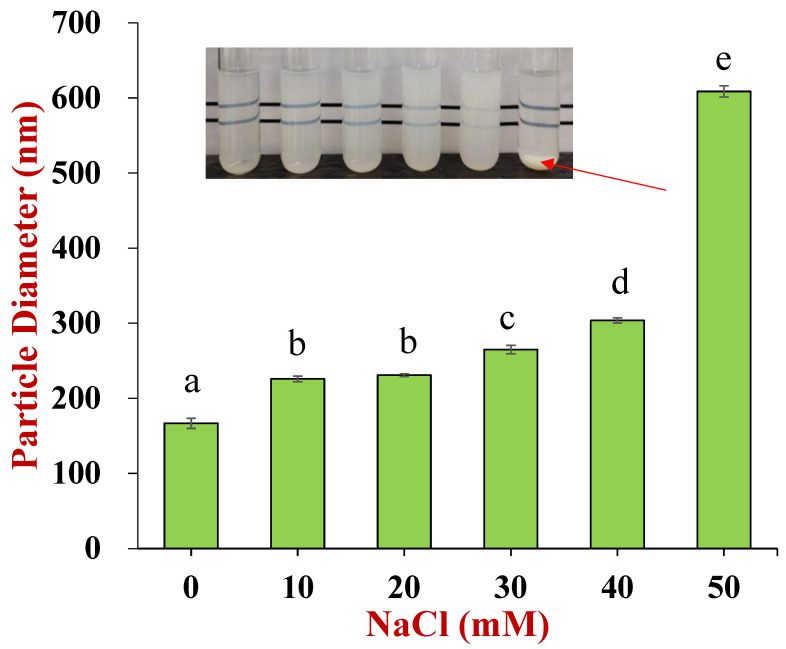
Effect of NaCl concentrations on nanoparticle stability. The inserted photo indicates that the appearance of nanoparticle dispersions changed with NaCl concentrations ranging from 0 to 50 mM (the letter “a–e” represents a statistically significant difference between two groups, *p* < 0.05).

The dependences of the zeta potential of the nutraceutical-loaded pectin-coated nanoparticles and pure pectin on pH are shown in Figure 4. The nanoparticles were negatively charged throughout the investigated pH range and had zeta potential values that were fairly similar to that of pure pectin. This result suggests that the nanoparticles were coated with pectin because the surface potential measurements are dominated by the electrical characteristics of the particle exteriors. The pectin molecules adsorb to the zein nanoparticle surfaces mainly through electrostatic attraction [14]. This phenomenon may account for the observed increase in mean particle diameter from 166.8 to 264.9 nm when the salt concentration was increased from 0 to 40 mM NaCl, as well as for the precipitation of the nanoparticles observed at 50 mM NaCl (Figure 5). The addition of salt ions weakened the electrostatic attraction between the anionic groups on the pectin molecules and the cationic groups on the zein nanoparticles through electrostatic screening effects. As a result, the pectin molecules became progressively detached from the nanoparticle surfaces, which led to bridging effects and a reduction in protection [43].

**Figure 6:**
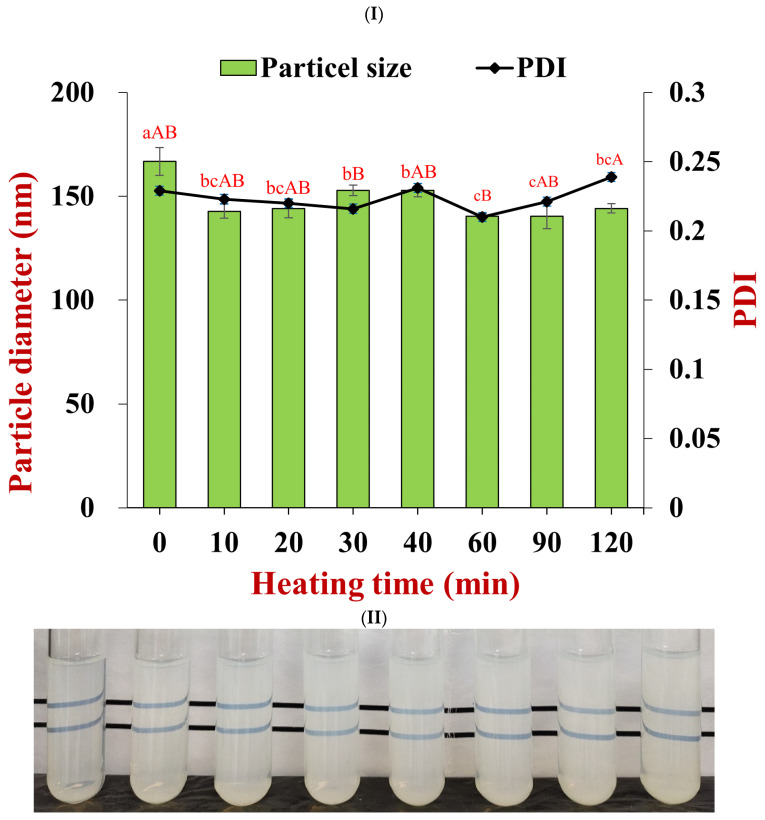
Particle stabilities after heating at 80 °C for 2 h. (**I**) particle diameter and PDI at different heating times (lowercase statistic notation—particle size; capital statistic notation—PDI, *p* < 0.05); (**II**) the appearance of particles after heat treatment.

The heat stability of the nanoparticle dispersions was assessed by heating them at 80 °C for 120 min. The average diameter of the nanoparticles decreased from 166.8 to 144.2 nm, while the polydispersity index remained small (0.21 to 0.24) after heating for 2 h (Figure 6I). The nanoparticle dispersions also remained transparent after being heated, even up to periods of 120 min (Figure 6II). These results suggest that the pectin-coated nanoparticles had a good resistance to heat stresses, which can again be attributed to the strong electrostatic and steric repulsion resulting from the polysaccharide coating [4]. 

The long-term stability of the nanoparticle dispersions was assessed by storing them at 4 °C for 28 days. There were no significant differences in the particle size and PDI values between the freshly prepared nanoparticles and those stored for 28 days (*p* > 0.05, Table 1), which indicated that the nanoparticles showed good storage stability.

In summary, the good pH, heat, and storage stabilities mean that these nanoparticles may meet the requirements for some commercial applications. However, the tendency for the nanoparticles to aggregate at modest ionic strengths may limit their application in products containing appreciable levels of salt.

### 3.3. In Vitro Antioxidant Activities of Co-Encapsulated Nanoparticles

Free radicals are present within the human body and in foods. They can react with lipids, phospholipids, proteins, and nucleic acids, altering their biological activities. The presence of high levels of free radicals in the body has been linked to several degenerative diseases, as well as the aging process. For this reason, we characterized the antioxidant activities of the nutraceutical-loaded nanoparticles using DPPH· and ABTS^+^· radical scavenging methods [27]. 

**Figure 7:**
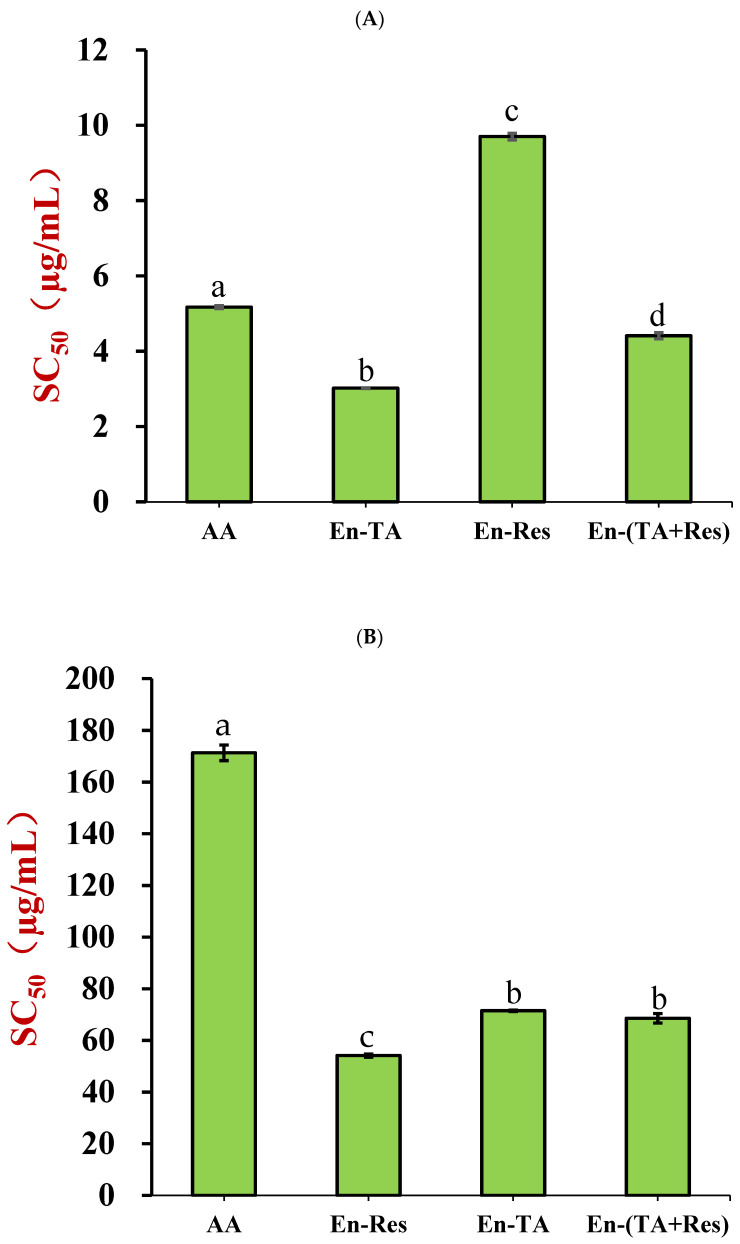
Comparison of the DPPH·(**A**) and ABTS^+^· (**B**) radical scavenging capacities (SC_50_ values) of nanoparticle co-encapsulated tannic acid and resveratrol (En-(TA+Res)), nanoparticle encapsulated tannic acid (En-TA), nanoparticle encapsulated resveratrol (En-Res), and the physical mixture of tannic acid and resveratrol (PM-(TA+Res)) with the same composition with En-(TA+Res) (the letter “a–d” represents a statistically significant difference between two groups, *p* < 0.05).

The DPPH· scavenging capacities (expressed as SC_50_) of tannic acid-, resveratrol-, and dual nutraceutical-loaded nanoparticles were compared to that of ascorbic acid (Figure 7A). In these experiments, a low SC_50_ value corresponds to a more potent antioxidant. The SC_50_ value of the nanoparticles containing only tannic acid was 3.02 μg/mL, which was significantly lower than that of ascorbic acid (5.17 μg/mL). This meant that the encapsulated tannic acid had a stronger radical scavenging capacity than ascorbic acid, which is known to be an effective natural antioxidant. Tannic acid-chitosan conjugate was also reported to have an SC_50_ of 4.3 μg/mL, which was comparable with our result [30]. In contrast, the radical scavenging capacity of the resveratrol-loaded nanoparticles (SC_50_ = 9.70 μg/mL) was almost two-fold less potent than that of ascorbic acid, and was also remarkably lower than that of free resveratrol, with a reported SC_50_ of 35.81 μg/mL [47]. The SC_50_ values of the nanoparticle containing both tannic acid and resveratrol was 4.41 μg/mL, which approached the SC_50_ value of the encapsulated tannic acid. The calculated weight ratio of tannic acid to resveratrol in the nanoparticles was 0.55:1, which meant that some synergetic effect of the DPPH· scavenging capacity existed in co-encapsulated tannic acid and resveratrol in the nanoparticles, and tannic acid increased the antioxidant capacity of resveratrol. The plain nanoparticles (no polyphenol loaded) only exhibited very weak radical scavenging capacity (Figure S1), which meant that the radical scavenging capacity could be attributed to the encapsulated polyphenols.

There was no difference of ABTS^+^· scavenging capacity between encapsulated tannic acid and co-encapsulated tannic acid and resveratrol (Figure 7B, *p* < 0.05), but encapsulated resveratrol exhibited a stronger scavenging capacity than that of the combined system. This suggested that there was no synergetic effect between tannic acid and resveratrol in scavenging ABTS^+^·. Ascorbic acid was not an effective antioxidant for scavenging ABTS^+^·, which is possibly due to its poor stability in PBS buffer at pH 7.4 [48]. Plain nanoparticles also exhibited a negligible radical scavenging capacity (Figure S2).

### 3.4. Effects of Co-Encapsulated Polyphenols on Peroxide-Induced Oxidative Stress in HepG2 Cells

Reactive oxygen species (ROS), such as hydrogen peroxide, hydroxyl free radicals, superoxide radicals, and singlet oxygen, are the products of normal cellular metabolism [39]. Moderate levels of ROS are required to regulate cell survival, but excessive levels induce oxidative stress in cells [39]. Hydrogen peroxide (H_2_O_2_) is a common reactive oxygen species in nature. For this reason, it is frequently used to promote oxidative stress in the cell culture models used to determine the antioxidant capacity of bioactive compounds, such as HepG2 cells [49,50]. Therefore, the cellular antioxidant activity of the co-encapsulated polyphenols was evaluated by measuring the MDA concentration and antioxidant enzyme activities (SOD and CAT) in peroxide-stressed HepG2 cells.

#### 3.4.1. HepG2 Cell Cytotoxicity

**Figure 8:**
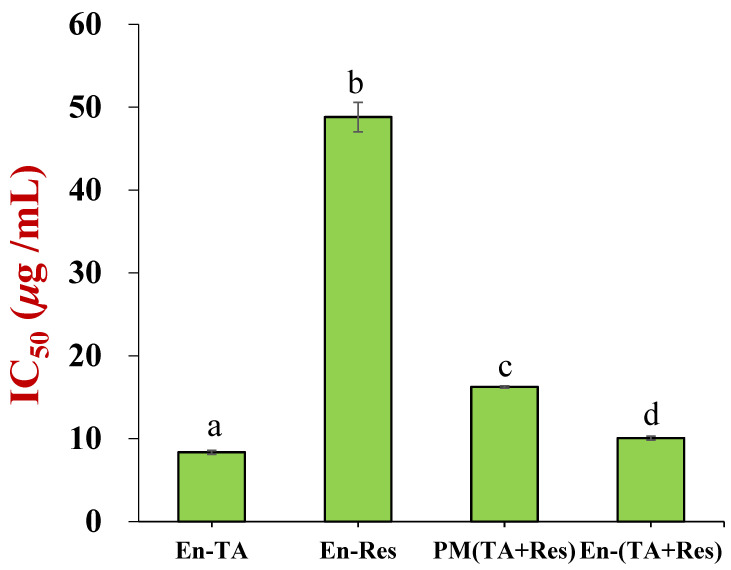
The HepG2 cell toxicity (IC_50_) of encapsulated tannic acid, resveratrol, and co-encapsulated tannic acid and resveratrol (the letter “a–d” represents a statistically significant difference between two groups, *p* < 0.05).

The effects of the concentration–effect relationships of encapsulated tannic acid and/or resveratrol, as well as a physical mixture of tannic acid, resveratrol, zein, and pectin with the same weight ratio as the co-encapsulated nanoparticles, on HepG2 cell viability were measured (Figures S3–S6). HepG2 cell viability decreased with increasing polyphenol concentration, which meant that tannic acid and resveratrol exhibited anti-proliferation effects on the cells. The cytotoxicity (IC_50_ values) of these formulations is shown in Figure 8. The encapsulated tannic acid was the most toxic to the HepG2 cell, with an IC_50_ value of 8.36 ± 0.22 μg/mL, which was 6-times lower than that of the encapsulated resveratrol (48.8 ± 1.8 μg/mL). The IC_50_ value of the co-encapsulated tannic acid and resveratrol was 10.07 ± 0.23 μg/mL, which approached that of the encapsulated tannic acid. It is interesting to note that the physical mixture containing water soluble tannic acid also exhibited high cell toxicity, with an IC_50_ value of 16.25±0.13 μg/mL. As shown in Figure S7, plain particles were nontoxic to HepG2 cells. Based on the cytotoxicity of these formulations, 4.2 µg/mL total polyphenol concentration was used to study the antioxidant capacity of the co-encapsulated tannic acid and resveratrol nanoparticles in the HepG2 cells.

#### 3.4.2. MDA Concentration and Antioxidant Enzyme Activity

**Figure 9:**
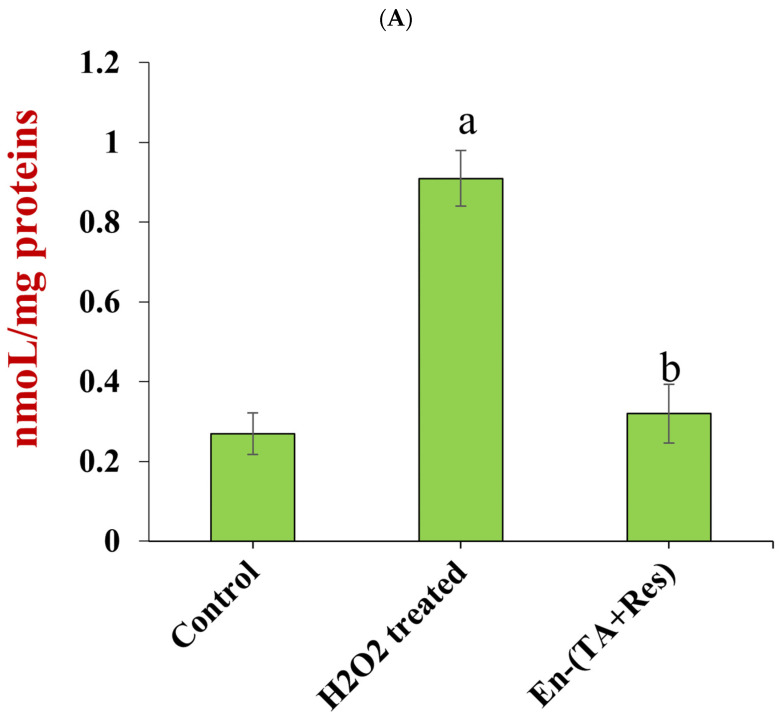

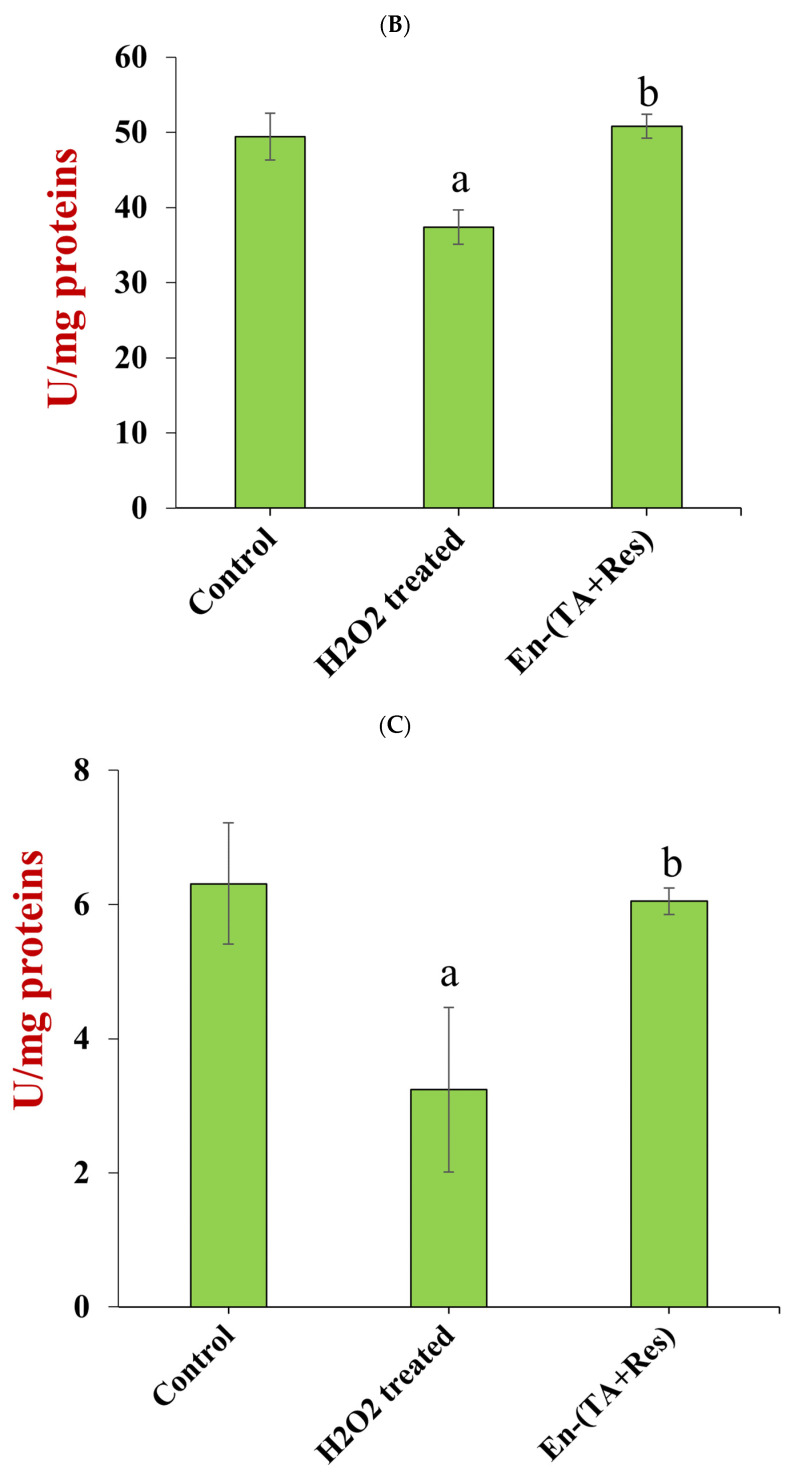
Effect of co-encapsulated tannic acid and resveratrol (En-(TA+Res)) on the concentration of MDA (**A**), activity of SOD (**B**), and activity of CAT (**C**) in HepG2 cells treated with H_2_O_2_ (the letter “a,b” represents a statistically significant difference between two groups, *p* < 0.05).

HepG2 ells were treated with 950 µmol/L H_2_O_2_ for 4 h to induce oxidative stress, and the cell survival rate was found to be around 50.3%. The MDA concentration in the H_2_O_2_ treated cells was 0.91 ± 0.07 nmol/mg proteins (Figure 9A), which was 3.4-times higher than that in normal HepG2 cells (Control = 0.27 ± 0.05 nmol/mg proteins). MDA is one of the main products of lipid peroxidation. It can react with proteins and nucleic acids, which can have adverse physiological consequences, including mutagenic and cytotoxic effects. Since the MDA concentration has been found to be elevated in various diseases related to free radical damage, it has been widely used as an index of lipoperoxidation in biological and medical sciences [51]. The increased MDA concentration in the peroxide-treated HepG2 cells indicated that H_2_O_2_ caused oxidative stress and lipid oxidation in the cells. The MDA concentration in the cells treated with co-encapsulated polyphenols was only 0.32 ± 0.07 nmol/mg proteins, which was 3-times lower than that of the peroxide-treated cells (*p* < 0.05), and almost approached that of the control. These results showed that the co-encapsulated tannic acid and resveratrol efficiently inhibited lipid oxidation in the peroxide-treated cells. 

The enzyme activities of SOD (Figure 9B) and CAT (Figure 9C) were inhibited by the peroxide treatment of the HepG2 cells, which decreased by 24.4% and 48.6% compared to the controls, respectively (*p* < 0.05). The oxidative stress induced by H_2_O_2_ is normally detoxified by the enzymatic antioxidant defense system. SOD catalyzes the reduction of the superoxide anion into hydrogen peroxide; then hydrogen peroxide and the other peroxides can be reduced by CAT. These enzymes are therefore actively involved in the antioxidant response of biological systems, and increased activities are indicative of oxidative stress [52]. The SOD and CAT activities in oxidative stress model cells treated with co-encapsulated polyphenols were 50.80 ± 1.85 U/mg proteins and 6.05 ± 0.20 U/mg proteins, respectively, which were significantly higher than those of H_2_O_2_ treated cells (*p* < 0.05). This suggested that co-encapsulated tannic acid and resveratrol succeeded in protecting the antioxidant enzyme activities from H_2_O_2_ damage.

### 3.5. Tannic Acid and Resveratrol Release under Simulated Gastrointestinal Conditions

**Figure 10:**
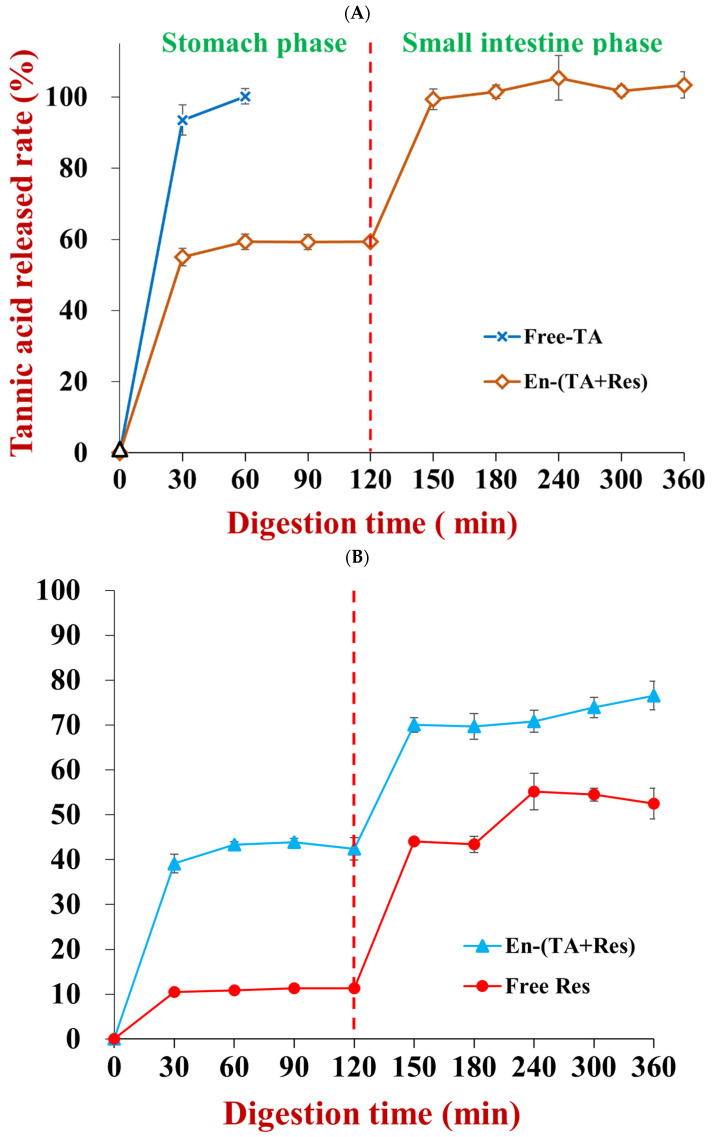
The releasing behavior of tannic acid (**A**) and resveratrol (**B**) from co-encapsulated nanoparticles during in vitro simulated gastric and small intestinal digestion.

The in vitro bioaccessibility of the co-encapsulated nutraceuticals was assessed by determining the percentage of tannic acid and resveratrol released into the simulated digestion fluids over time. Since tannic acid is soluble in water, free tannic acid was totally released in one hour during stimulated stomach digestion (Figure 10A). Conversely, the release of tannic acid occurred more slowly when it was encapsulated in the nanoparticles. Indeed, only about 59.4% of the tannic acid was released into the gastric fluids after 120 min digestion [28]. This value was about two-fold higher than when tannic acid alone was encapsulated in zein nanoparticles [28], which suggests that the presence of the resveratrol may have impacted the release of the tannic acid under gastric conditions. At the small intestine phase, the tannic acid remaining in the nanoparticles was totally released within the first 30 min of digestion. This value was also higher than the value (about 64%) reported when tannic acid was encapsulated alone in zein nanoparticles [28]. These results show that co-encapsulation with resveratrol increased tannic acid release during stimulated digestion. 

The percentage of encapsulated resveratrol released (42.4%) was about 4-fold higher than that of non-encapsulated resveratrol (11.3%) after 120 min of stomach digestion. The resveratrol continued to be released in the small intestine for both systems, but the differences between them were much less noticeable than those in the stomach. Even so, by the end of the small intestine phase, the total percentage of encapsulated resveratrol released (76.6%) was still appreciably higher than that of the non-encapsulated resveratrol (52.5%). Comparing our results with previous studies on resveratrol-loaded zein nanoparticles, it seems that co-encapsulating resveratrol with tannic acid did not impact its release [39]. Indeed, it was previously reported that the percentages of resveratrol released from resveratrol-loaded zein nanoparticles after stomach and small intestine digestion were 37.0% and 77.1%, respectively.

## 4. Conclusions

In this study, we used pectin-coated zein nanoparticles to co-encapsulate a hydrophobic (resveratrol) and hydrophilic (tannic acid) nutraceutical. The nutraceutical-loaded nanoparticles created by antisolvent precipitation and electrostatic deposition exhibited a relatively small size (166.8 nm) and low polydispersity index (0.25). Moreover, they showed relatively high encapsulation efficiencies for both tannic acid (around 51.5%) and resveratrol (around 77.2%). The nutraceutical-loaded nanoparticles were stable from pH 2.0 to 8.0 when stored at 4 °C for 28 days and when heated at 80 °C for 2 h, which may be important for some commercial applications. On the other hand, they tended to aggregate in the presence of salt, which was attributed to a weaking of the electrostatic attraction between the pectin coating and zein nanoparticles. The co-encapsulated tannic acid and resveratrol exhibited strong in vitro antioxidant activity and were shown to decrease the oxidative stress in a cell culture model, which was indicated by significantly decreasing the malondialdehyde (MDA) concentration and increasing the superoxide dismutase (SOD) and catalase (CAT) activities in peroxide-treated human hepatoma cells (HepG2). The encapsulation of the nutraceuticals also changed their release characteristics under simulated gastrointestinal conditions. In the stomach, encapsulation inhibited the release of tannic acid, but promoted the release of resveratrol. However, in the small intestine, it led to a relatively high bioaccessibility of both quercetin and tannic acid. Our results suggest that colloidal delivery systems can be produced from zein nanoparticles that contain both hydrophilic and hydrophobic nutraceuticals or drugs. Future research should focus on understanding the in vivo retention, release, absorption, and bioavailability of tannic acid and resveratrol throughout the gastrointestinal tract using animal models.

## Figures and Tables

**Table 1:** The mean size, PDI, and ζ-potential of the nanoparticles after long-term storage.

Storage Days	Mean Size (nm)	PDI	ζ-Potential (mV)
0	166.8 ± 6.75 ^a^	0.229 ± 0.006 ^a^	−26.0 ± 1.72 ^a^
7	167.0 ± 6.96 ^a^	0.220 ± 0.016 ^a^	−27.5 ± 0.29 ^a^
28	162.4 ± 8.68 ^a^	0.216 ± 0.007 ^a^	−26.5 ± 0.60 ^a^

Note: Lowercase letters in the same column indicate no significant differences according to the Dunnett test at the *p* < 0.05 level.

## Data Availability

The data are available from the corresponding author.

## Supplementary Materials

The following are available online at https://www.mdpi.com/article/10.3390/foods11213478/s1, Figure S1: The DPPH∙ scavenging capacity of plain nanoparticles. The concentrations of plain nanoparticles corresponded to tannic acid and resveratrol co-encapsulated nanoparticles. Figure S2: The ABTS^+^∙ scavenging capacity of plain nanoparticles. The concentrations of plain nanoparticles corresponded to tannic acid and resveratrol co-encapsulated nanoparticles. Figure S3: The viability of HepG2 cells treated with nanoparticle encapsulated resveratrol for 24 h. Figure S4: The viability of HepG2 cells treated with nanoparticle encapsulated tannic acid for 24 h. Figure S5: The viability of HepG2 cells treated with nanoparticle co-encapsulated tannic acid and resveratrol for 24 h. Figure S6: The viability of HepG2 cells treated with tannic acid and resveratrol physical mixture for 24 h (the mixture had the same compositions as the co-encapsulated nanoparticles). Figure S7: The viability of HepG2 cells treated with plain nanoparticles for 24 h. The concentrations of plain nanoparticles corresponded to tannic acid and resveratrol co-encapsulated nanoparticles (*n* = 3, *p* > 0.05).
