# Supplementary material for: Co-Encapsulation of Tannic Acid and Resveratrol in Zein/Pectin Nanoparticles: Stability, Antioxidant Activity, and Bioaccessibility
Source: Foods. 2022 Nov 2;11(21):3478. doi: 10.3390/foods11213478 (PMC9656218; doi:10.3390/foods11213478)
Supplement: Supplementary file 1 [file foods-11-03478-s001.zip › foods-1993826-supplementary.pdf]

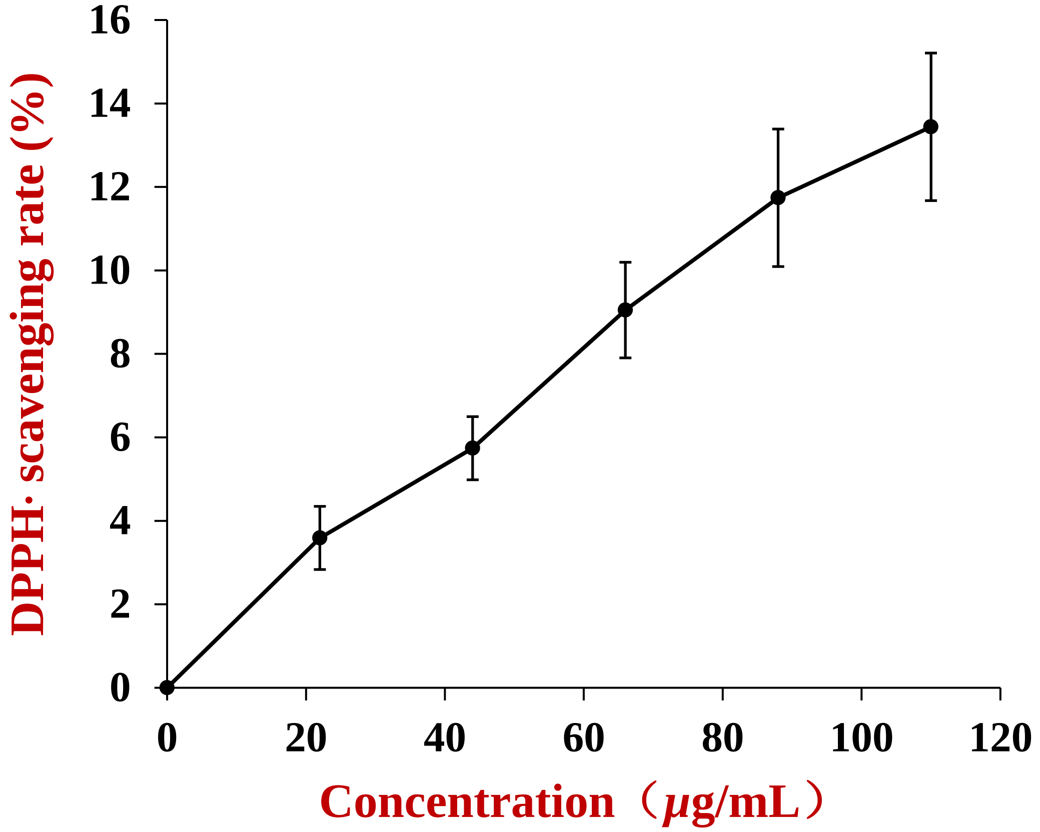

Figure S1. The DPPH· scavenging capacity of plain nanoparticles. The concentrations of plain nanoparticles were corresponding to tannic acid and resveratrol co-encapsulated nanoparticles.

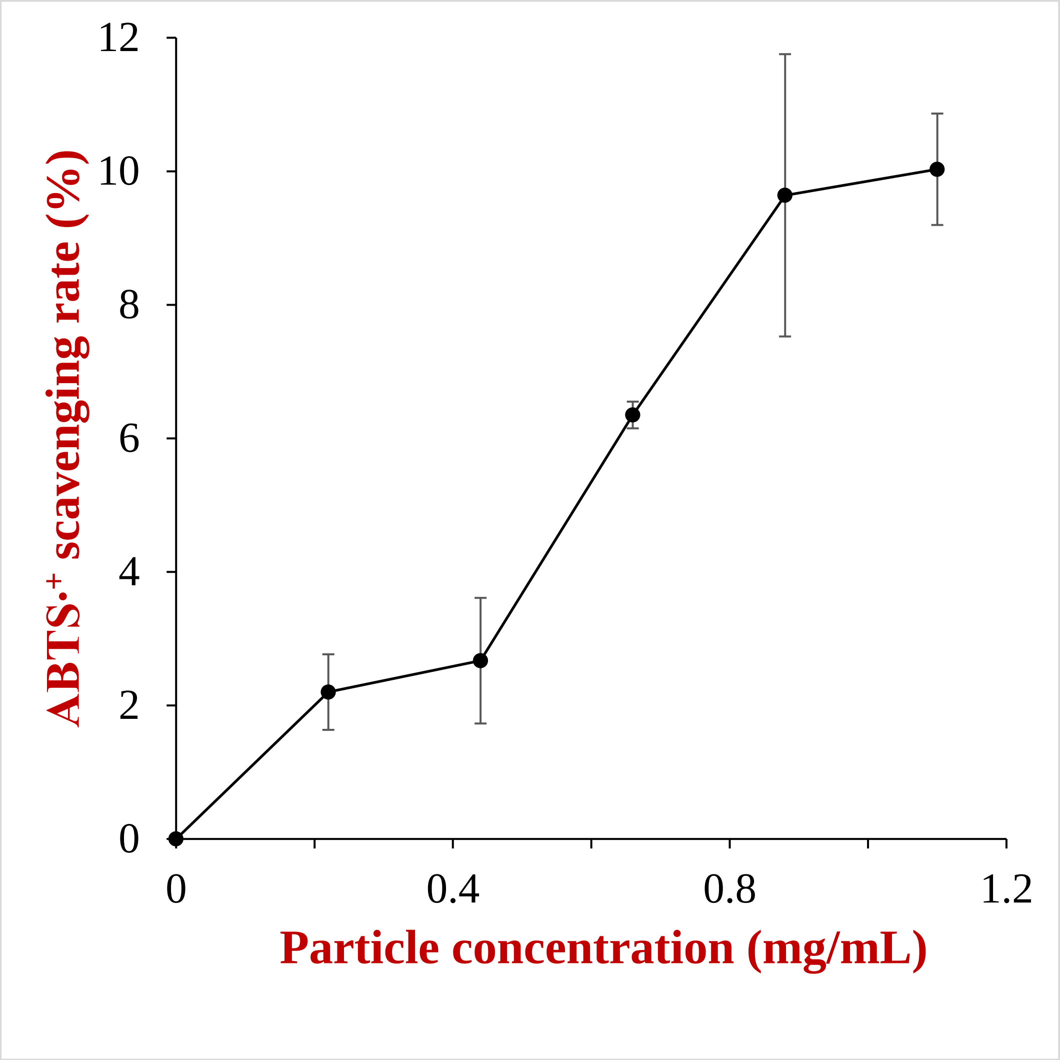

Figure S2. The ABTS$^{+\cdot}$ scavenging capacity of plain nanoparticles. The concentrations of plain nanoparticles were corresponding to tannic acid and resveratrol co-encapsulated nanoparticles.

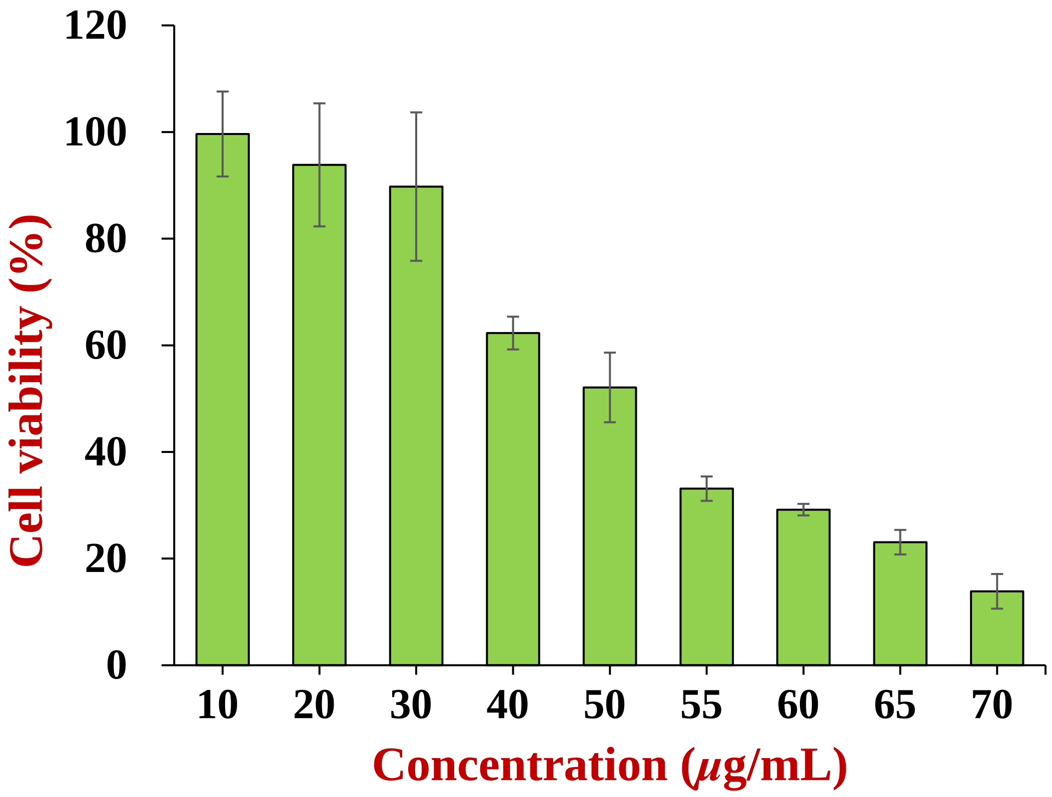

Figure S3. The viability of HepG2 cells treated with nanoparticle encapsulated resveratrol for 24 h.

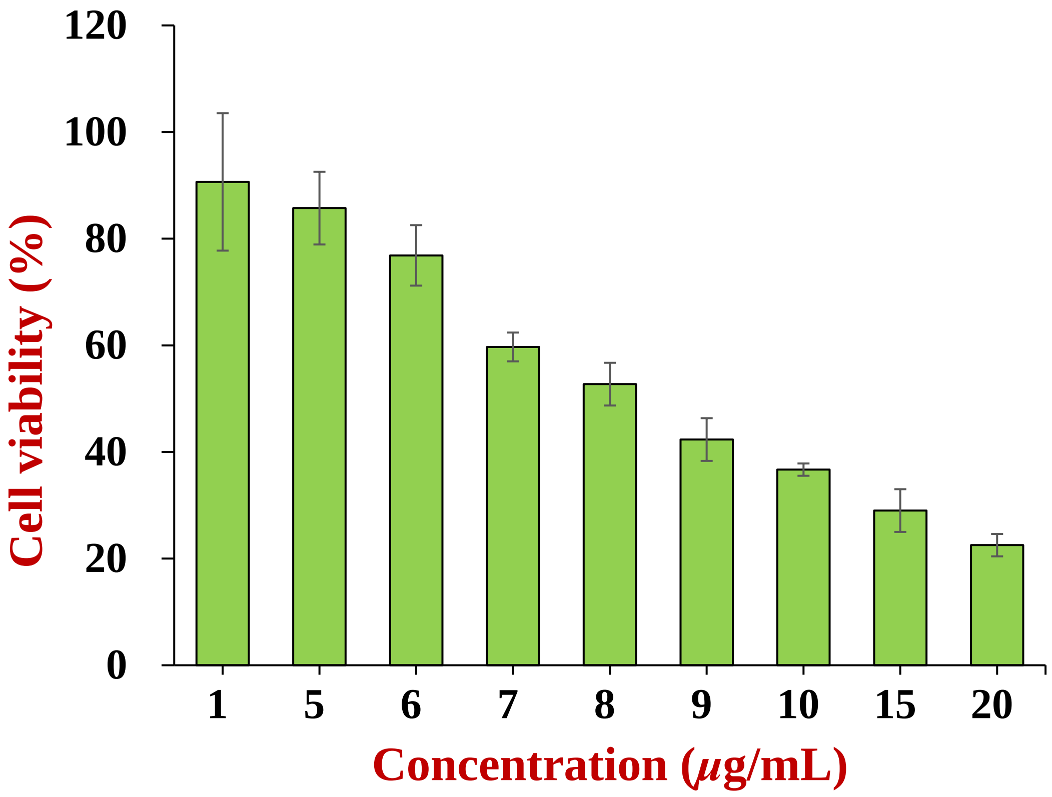

Figure S4. The viability of HepG2 cells treated with nanoparticle encapsulated tannic acid for 24 h.

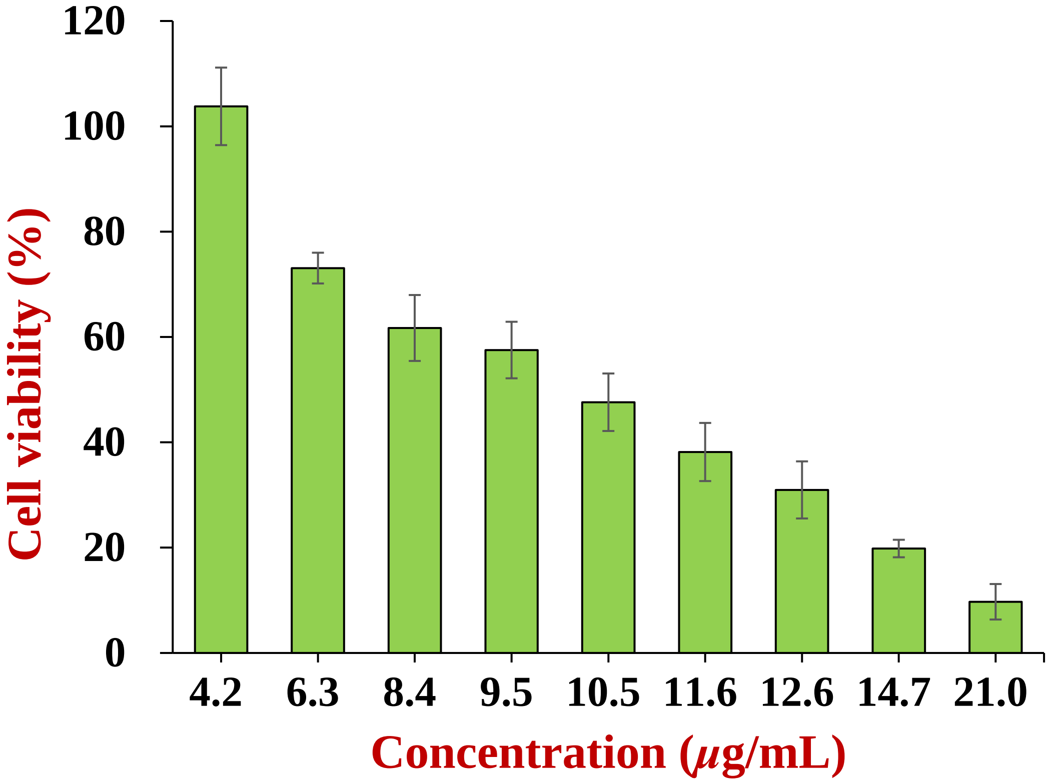

Figure S5. The viability of HepG2 cells treated with nanoparticle co-encapsulated tannic acid and resveratrol for 24 h.

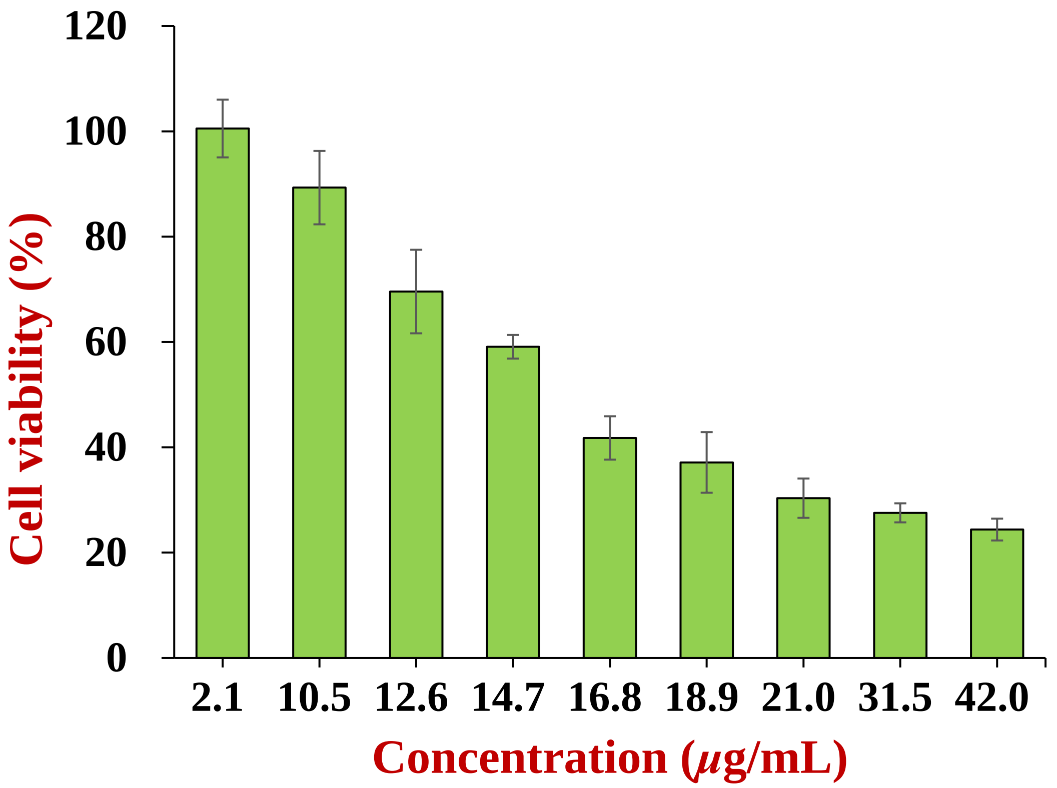

Figure S6. The viability of HepG2 cells treated with tannic acid and resveratrol physical mixture for 24 h (the mixture had the same compositions to co-encapsulated nanoparticles).

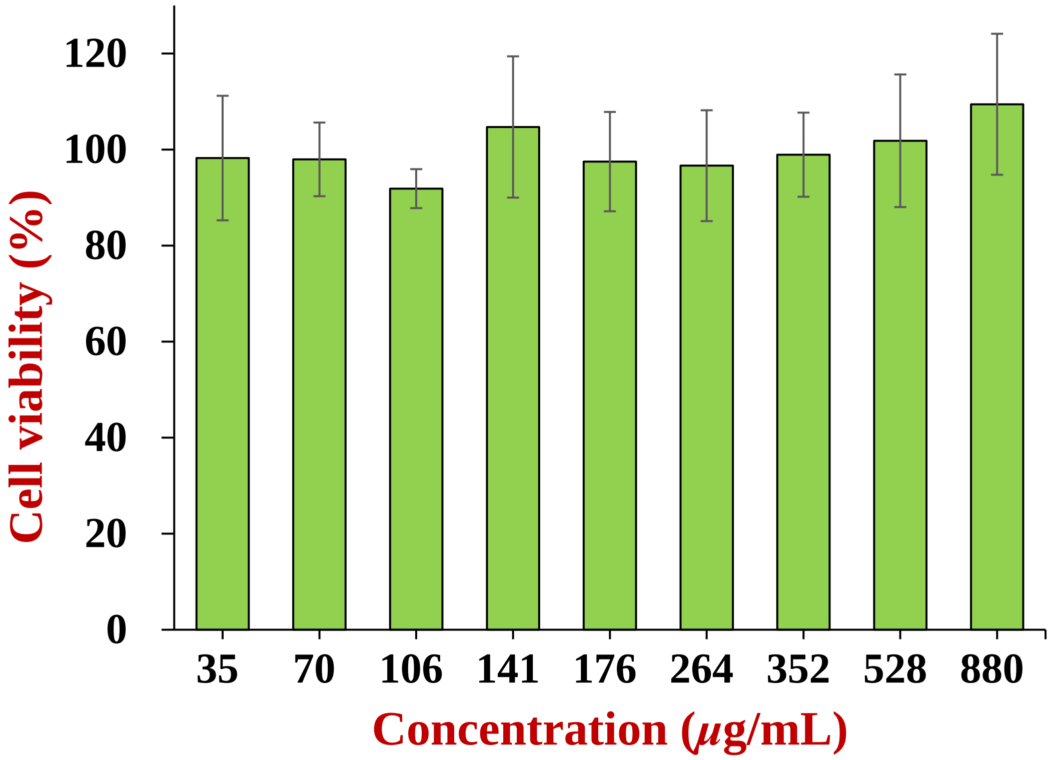

Figure S7. The viability of HepG2 cells treated with plain nanoparticles for 24 h. The concentrations of plain nanoparticles were corresponding to tannic acid and resveratrol co-encapsulated nanoparticles (n=3, p>0.05)
